# “Thinking a Lot” Among the Khwe of South Africa: A Key Idiom of Personal and Interpersonal Distress

**DOI:** 10.1007/s11013-015-9475-2

**Published:** 2015-10-20

**Authors:** T. N. den Hertog, M. de Jong, A. J. van der Ham, D. Hinton, R. Reis

**Affiliations:** Amsterdam Institute for Social Science Research, University of Amsterdam, Postbus 15718, 1001 NE Amsterdam, The Netherlands; African Studies Centre, Leiden, The Netherlands; Reha-Prime, Rehabilitation and Physiotherapy, Zurich, Switzerland; Athena Institute for Research on Innovation and Communication in Health and Life Sciences, VU University, Amsterdam, The Netherlands; Massachusetts General Hospital, Harvard Medical School, Boston, MA USA; Department of Public Health & Primary Care, Leiden University Medical Center, Leiden, The Netherlands; School of Child and Adolescent Health, the Children’s Institute, University of Cape Town, Cape Town, South Africa

**Keywords:** Mental health, Thinking too much, Idioms of distress, South Africa, San

## Abstract

“Thinking too much”, and variations such as “thinking a lot”, are common idioms of distress across the world. The contextual meaning of this idiom of distress in particular localities remains largely unknown. This paper reports on a systematic study of the content and cause, consequences, and social response and coping related to the local terms *|x’an n|a te* and *|eu*-*ca n|a te*, both translated as “thinking a lot”, and was part of a larger ethnographic study among the Khwe of South Africa. Semi-structured exploratory interviews with community members revealed that “thinking a lot” refers to a common experience of reflecting on personal and interpersonal problems. Consequences were described in emotional, psychological, social, behavioral, and physical effects. Coping strategies included social support, distraction, and religious practices. Our contextualized approach revealed meanings and experiences of “thinking a lot” that go beyond a psychological state or psychopathology. The common experience of “thinking a lot” is situated in socio-political, economic, and social context that reflect the marginalized and displaced position of the Khwe. We argue that “thinking a lot” and associated local meanings may vary across settings, may not necessarily indicate psychopathology, and should be understood in individual, interpersonal, community, and socio-political dimensions.

## Introduction

This paper reports on a study that explored meanings associated with local idioms referring to excessive thinking (e.g., “thinking a lot”) among a group of Khwe living in South Africa. These idioms were identified and came up frequently in previous studies conducted among the Khwe (Den Hertog forthcoming).

Idioms of excessive thinking, often referred to as “thinking too much”, are described across the world and are commonly discussed in relation to cultural concepts of mental illness or psychological distress. Patel, Simunyu, and Gwanzura (1995) in Zimbabwe were one of the first to describe “thinking too much” in academic literature. The authors describe the Shona term *Kufungisisa* (“thinking too much”) as a local causal explanation and symptom of emotional or psychological illness. The *Diagnostic and Statistical Manual of Mental Disorders* (DSM-5) includes “thinking too much”, sub-headed as “Kufungisisa”, as an idiom of distress and causal explanation used in various cultural contexts and world regions (American Psychiatric Association 2013). Kaiser et al. (2014) note that over 130 studies in diverse world regions have reported on “thinking too much”. Few of these studies were designed to systematically study the concept; generally, “thinking too much” was one of many cultural concepts reported upon. A review of more systematic studies revealed several possible characterizations for the expression. First, “thinking too much” reflects a cognitive process of ruminating about current life problems (Hinton et al. 2015; Kaiser et al. 2014; Patel, Simunyu, and Gwanzura 1995; Yarris 2014), sad events of the past (e.g. losing a loved one or traumatic event) (Hinton et al. 2015), or minor things (Yang et al. 2010). Second, “thinking too much” has several associated symptoms including: emotional affects such as anxiety, worry, sadness (Hinton et al. 2015; Kaiser et al. 2014; Patel, Simunyu, and Gwanzura 1995; Yang et al. 2010; Yarris 2014); somatic complaints (e.g. severe headache) (Hinton et al. 2015; Patel, Simunyu, and Gwanzura 1995; Yarris 2014); and social consequences such as social withdrawal (Kaiser et al. 2014; Patel, Simunyu, and Gwanzura 1995). Third, “thinking too much” is considered as a cause of severe conditions or phenomena such as psychotic illness, or ‘insanity’ (Hinton et al. 2015; Kaiser et al. 2014; Yang et al. 2010). Finally, “thinking too much” has a communicative function to express the experience of distress (Yarris 2014).

Hinton et al. (2015) have identified five important areas of attention for exploring the contextual meaning of “thinking too much”: (1) content of “thinking too much” to address the ecological context and source of distress, (2) the induced symptoms, (3) the semantic context, (4) the role in psychopathological processes, and (5) local treatments and coping. The article by Hinton et al. (2015), and the others reviewed, suggest the need for a systematic, multi-dimensional examination of idioms of distress in sociocultural context.

This paper reports on such a contextualized investigation of “thinking a lot” as an “idiom of distress” in a particular group and location. Nichter (1981) coined the term “idiom of distress” and used it to refer to cultural concepts that communicate distress, and he emphasized the localized meaning and social ramifications of an idiom in its socio-cultural context. During the 30 years since the term “idiom of distress” was introduced, it has at times been used to describe an illness category or “cultural syndrome” (de Jong and Reis 2010; Nichter 2010). Nichter believes that idioms of distress should primarily be understood as modes of experiencing and expressing distress (1981, 2010). In contrast, (cultural) syndromes are linked to a specific set of symptoms (American Psychiatric Association 2013; Nichter 2010) acknowledged by local practitioners as prototypical ailments, or types of behavior locally recognized as types of distress (Nichter 2010). A syndrome may also function as idiom of distress (Nichter 2010). Idioms of distress are not necessarily indicative of psychopathological distress states. The personal or social suffering that idioms of distress communicate vary from mildly stressful experiences to severe distress states that disrupt the daily functioning of individuals and groups (American Psychiatric Association 2013; Nichter 2010). Rather than considering idioms of distress as mere identifiers of underlying experiential states, studying idioms of distress in a socio-political and cultural context allows us to gain insight into underlying meanings. For example, idioms of distress may identify interpersonal, social, economic, political, and spiritual sources of distress (Nichter 2010), or reveal why these modes of expression are appropriate (de Jong and Reis 2010; Nichter 1981, 2010). Idioms of distress may also help us to understand experiences of distress in relation to ethnophysiology and ethnopsychology (Good 1977). Ignoring the socio-political and cultural context of idioms of distress has been referred to as “an error of decontextualization” (Hinton and Good 2015). For example, only determining how “thinking too much” relates to Western psychiatric illnesses or symptoms strips the expression of its contextual meaning—its position within local systems of meanings and realities. In this study, we aimed to achieve a contextualized understanding of the idiom of distress “thinking a lot” among the Khwe of South Africa. We focused particularly on the content and cause of “thinking a lot”, the consequences, and the Khwe’s social responses and coping.

### The Socio-cultural and Historical Context of the Study

The Khwe reside together with the !Xun^1^ in a township, Platfontein, on the outskirts of Kimberley, the capital city of the Northern Cape province of South Africa. The Khwe and !Xun are two San or Bushman^2^ communities of approximately 1700 and 4500 people, respectively (South African San Institute 2010). The San are often described as the indigenous people of southern Africa and have been positioned on the bottom of the local social hierarchy throughout history. At times, the San have even been considered to be more like an animal than a man or woman and treated as vermin (Gordon and Sholto-Douglas 2000). Similar to other indigenous groups, the San are a cultural and linguistic minority. The Khwe and !Xun predominantly speak a distinct and mutually unintelligible San language, Khwe and !Xun, in everyday interactions within their community. They rely on Afrikaans as a lingua franca and some speak English. Both Khwe and !Xun are primarily spoken languages since these languages are rarely written. Khwe is a dynamic and evolving language. This is partly due to the fact that the Khwe community includes Khwe who originate from various regions with minor differences in language use. In addition, exposure to Afrikaans and English words, for which there are no equivalents in Khwe, has resulted in the invention of new Khwe words or a modification and inclusion of Afrikaans and English words into the Khwe language.

The Khwe and !Xun are originally from southern Angola and northeast Namibia and came together in a history of war and displacement (Den Hertog 2013). They were involved in two interrelated wars: the Angolan War of Independence and the South African Border War. The Khwe and !Xun fought alongside the Portuguese and the South African Defence Force (SADF). After Angola’s independence in 1975, many Khwe and !Xun fled the country in fear of retribution by former enemies. This was not unfounded considering the local population’s wish for revenge on the San and reports of a large number of San killed during and near the end of the war (Battistoni and Taylor 2009; Brinkman 2005:120–121; Robbins 2007; South African San Institute n.d.). Many Khwe and !Xun ended up in the Caprivi area in Namibia, bordering both Angola and Botswana, and were incorporated into the SADF as were local Khwe in the Caprivi area. The Khwe and !Xun became dependent on the SADF since it became an integral part of their everyday life for employment and everyday activities, schooling, and provision of general services (Gordon and Sholto-Douglas 2000). Following Namibian independence in 1990, many of the Khwe and !Xun came to South Africa out of loyalty to the SADF, hope for future compensation for their contribution in the South African Border War, and possible fear of retribution from the South West Africa People’s Organization (SWAPO) (South African San Institute n.d.). In South Africa, the Khwe and !Xun continued to live under the auspices of the SADF in a tented camp on a military base near the small town of Schmidtsdrift in the Northern Cape. Uncertainty for their future was increasingly problematic as the political climate changed from apartheid to a majority-rule democracy and the Khwe and !Xun had an identity as apartheid ‘mercenaries’ (Geldenhuys 2011:655–658; Sharp and Douglas 1996). The promised housing development was put aside, former ‘bushman’ battalions were disbanded, and job availability within the defense force decreased dramatically (Robbins 2006). Uncertainty further increased when a third forced relocation became imminent when a local community, the Batlhaping, successfully filed a land claim covering the land of the military base. At the end of 2003, and in early 2004, the Khwe and !Xun relocated to Platfontein.

A history of marginalization and displacement continues into the present as the Khwe and !Xun face a myriad of problems while trying to build a life in South Africa. Although their relocation to the township brought improvements, such as housing and a relative proximity to town, the housing’s poor quality and poor provision of services in comparison to neighboring communities fuelled feelings of marginalization and neglect by the local government (Tempelhoff 2014). In response to overcrowding in houses constructed in 2003 and absence of governmental action, people started constructing shanty houses. Poverty is one of the most pressing issues faced by the Khwe and !Xun communities where 97 % live on less than 1 US dollar/day and there is a structural lack of opportunities to improve life with an unemployment rate of 95 % (Dalton-Greyling and Greyling 2007; South African San Institute 2010). Many families rely on social benefits or income generated by one of the family members. In order to supplement rations some people plant peanuts, corn, and various Namibian vegetables. The few employment opportunities that are available are security and farm work, which require individuals (mostly men) to stay at the work location for consecutive periods of three to nine months. Community members cite disruptions in family life such as extramarital affairs and money disputes as being related to these work arrangements. Alcohol abuse among the Khwe and !Xun, as well as other San communities, is commonly reported (Robins, Madzudzo, and Brenzinger 2001) and indicative of dysfunctional community life. In addition, although statistics are not available, HIV/AIDS and tuberculosis are considered major health issues by staff at the local clinic, community leaders and local NGOs (Govender et al. 2013; Letsoalo 2010). These disruptive living conditions put continuous pressure on the social fabric of Khwe and !Xun communities and increase their vulnerability for experiencing distress.

Despite scholarly interest in San communities, there is a paucity of research on mental health and psychological well-being of this group; prevalence data on mental distress are unavailable. Although the communities’ marginalized position and disrupted social life are acknowledged, only minor remarks are made about the need for studies and interventions to address individual psychological well-being (e.g. Robins, Madzudzo, and Brenzinger 2001:22–23). Interventions for distress states are nearly absent. In Schmidtsdrift, psychological counseling was supposedly offered (Robbins 2006), but was most likely small-scale considering our informants’ poor memory of this service during our fieldwork. Currently, primary health care facilities in Platfontein and Kimberley provide mental health care services as part of the community-based mental health care policy (Ramlall 2012), but they are already overburdened and understaffed (Petersen and Lund 2011). To facilitate development of appropriate mental health interventions, research is needed to understand the experiences and sources of distress, and identify locally appropriate coping strategies. This study aims to contribute to an emic understanding of distress by reporting on “thinking a lot” as a key idiom of distress among the Khwe.

## Method

This study is part of an ethnographic doctoral study on mental health perceptions and care among the Khwe and !Xun that took place between 2012 and 2014. During three fieldwork visits (approximately three months each), the first author worked with a group of master students to conduct studies on mental health. Excessive thinking (viz., “thinking too much”, “thinking a lot”, and “thinking about many things”) came up frequently in these studies. In a pilot study on mental health perceptions (Den Hertog forthcoming), excessive thinking was described as causal explanation for ‘madness’. In a vignette study on depression, Den Hertog et al. (forthcoming) identified excessive thinking as a key dimension that connects life struggles to emotional affect. In daily interaction with community members, excessive thinking was sometimes used to indicate moments of distress and life struggles. To achieve a better understanding of this idiom of distress, we conducted a systematic follow-up study. Semi-structured interviews were conducted with Khwe community members that focused on the content and cause of “thinking a lot”, the consequences, and social responses and coping. In addition, ethnographic data was used to better delineate the meaning of “thinking a lot” in the local socio-cultural context.

### Data Collection and Analysis

To retain cultural and language meanings, we conducted the study in one community, the Khwe. This community was chosen because existing relationships allowed access to research facilitators, interpreters and there was an available Khwe-English dictionary (Kilian-Hatz 2003). We had none of these resources for working with the !Xun at the time of the fieldwork. Semi-structured exploratory interviews (Rubin and Rubin 2005) were conducted from March to June in 2014 by the first and second author. An interview guide was developed to ensure all themes of interest were discussed with respondents. Both the first and second authors were present during the first 12 interviews and each rotated the interviewer and observer role. Minor alterations were made to the interview guide to improve the flow of the interview and make questions more intelligible for respondents. The second author conducted the additional eight interviews alone or with a local research facilitator.

In the first research phase, we conducted interviews with four community members who had experience writing the Khwe language (e.g. by following a Khwe literacy workshop) and translation work (e.g. translating community meetings, or being a field worker for linguistic research projects). These respondents served as language advisors and were selected on the basis of information from our research facilitators and other informants from the Khwe community. The interviews served to elicit Khwe words associated with excessive thinking and explore general characteristics such as how and when the Khwe words are used, associated experiential states, content of excessive thinking, causal explanations, consequences, and social response and coping.

In the second research phase, we conducted interviews with general Khwe community members. In our sampling, we strived for a variety of age groups, education levels, employment status, and an equal distribution in gender. Community members were recruited by walking around the Khwe community and asking people to participate in the study. In addition, snowball sampling was used and local research facilitators assisted the recruitment of respondents that were under-represented our sample—specifically older respondents. The Khwe words elicited in the first phase were used to initiate and provide focus in second round of interviews. Respondents were asked to give a general description of the terms associated with excessive thinking and make free associations. This was followed by questions about translations of Khwe into Afrikaans and English. Thereafter the main focus of the interview concerned the content of “thinking a lot”, the use of the concept in social interactions, and key characteristics of explanatory models (EM) (Kleinman, Eisenberg, and Good 1978): symptoms, timeline and duration, causal explanations, consequences, and coping strategies. To gain additional depth in our data, we asked respondents whether they personally experienced excessive thinking and, if so, to reflect on these experiences.

In total, 20 interviews took place, including four interviews with language advisors. Interviews were conducted in the language preferred by respondents. Eight interviews were conducted in English, five in Afrikaans, one in a combination of Afrikaans and English, and six in Khwe. A bilingual Afrikaans-English speaker translated the English interview guides into Afrikaans. The first author, who fully comprehends Afrikaans and speaks it at a basic level, conducted the Afrikaans interviews. We relied on our research facilitators for the interpretation and translation of Khwe interviews. Debriefing was used to identify difficulties in translation and facilitate learning for future translations (Borchgrevink 2003). Despite the limitation of being unable to directly communicate with respondents in Khwe, respondents were able to express their views on excessive thinking with relative ease. Data analysis revealed a general consistency within themes, which indicates that we were able to elicit important meanings attributed to the Khwe terms. However, at times, responses remained superficial, which indicates that language barriers may have restrained the richness of some of our data.

Interview data was complemented by drawing on ethnographic data of living conditions, everyday activities, and common topics of discussion obtained by observations and ‘hanging-out’ during field visits (Angrosino 2007). Field notes and daily reflections were used to record observations and informal conversations.

Interviews were audio-recorded and transcribed verbatim. Transcripts and ethnographic data were analyzed using qualitative data-analysis software (Atlas.ti version 7). Descriptive coding was used to structure data and codes were derived from the interview topic list and created inductively (Miles and Huberman 1994). Results are presented in overarching themes to describe the full breadth of data and indicate key sub-themes.

### Ethical Considerations

Before the start of an interview, the research purpose and aim, interview process, audio-recording, and the rights concerning participation were verbally discussed with respondents and presented in writing. Respondents were then asked to sign an informed consent. Interviews took place at a location chosen by respondents. Ethical clearance was obtained from the Humanities & Social Sciences Research Ethics Committee of the University of KwaZulu-Natal (ref. number HSS/0054/013D)^3^.

## Results

A description of respondents’ characteristics is provided after which local meanings of “thinking a lot” among the Khwe are described in the following overarching themes: “thinking a lot” in three languages, general descriptions, content and causes, consequences, and coping.

### A Description of Respondents

In total, 20 respondents were interviewed including 9 women and 11 men. The majority of respondents (12) were below the age of 35. The other eight respondents varied between 35 and 79 years. Of our respondents, 2 did not receive any formal education, 4 dropped out before grade 10, 11 attended grades between grade 10 and 12, and 3 respondents continued education or training beyond grade 12. Eight of our respondents were unemployed or received social benefits, four were attending their final years in school, and eight were employed (most of them at the community radio station). Our sample was relatively young and more respondents were employed than could be expected from employment statistics.

### “Thinking a Lot” in Three Languages

Previous studies conducted among the Khwe and !Xun, described in the methods section, identified three terms for excessive thinking: *thinking too much* in English, and the Afrikaans terms *dink baie*, and *dink aan klomp goeters*. *Dink baie* is translated as “thinking a lot”.^4^*Dink aan klomp goeters* is translated as “thinking about a lot of things”.^5^

Language advisors identified the following three Khwe terms associated with the above-mentioned Afrikaans and English terms followed by a literal translation with a Khwe dictionary (Kilian-Hatz 2003): *|x’an n|a te*^6^ “much thinking”,^7^*|eu-can|a te* “big thinking”,^8^ and *tiya xo’a n|a te* “many things thinking”.^9^ Language advisors explained the meaning and use of the three Khwe terms as ruminating about life problems. The first two Khwe terms seemed more commonly used as became clear from their examples. We therefore focused our attention on these Khwe terms and used them to initiate interviews with general community members.

In our interviews, respondents recognized both common Khwe terms (*|x’an n|a te* and *|eu-can|a te*), and used them interchangeably. They did not distinguish the meanings of the two expressions and translated them into a single Afrikaans term, *dink baie* (“thinking a lot”). This indicates that *|x’an n|a te* and *|eu-can|a te* are expressions that refer to one set of meanings and associations, and from here on they will be referred to as “thinking a lot”. In light of this study, it is interesting to note that the concept of the English word “stress” and the Afrikaans equivalent of *stres* are commonly known and used, especially among the younger generation. “Stress” has no equivalent in Khwe and was occasionally used while speaking Khwe. “Stress” was sometimes used to refer to an experiential state associated with or caused by “thinking a lot”, and linked to current life problems. However, other respondents used the word “stress” interchangeably with “thinking a lot” or “thinking too much” and when asked about the difference, they mentioned that these terms referred to the same thing.

### General Descriptions

Respondents’ general descriptions and examples of situations in which *|x’an n|a te* and *|eu-can|a te* were used indicates that “thinking a lot” refers to an intense form of thinking and is distinguished from “normal” thinking: “In our language you hear people saying ‘ti |x’an n|a te’ (I think a lot), that he is thinking of something and he is not taking it lightly, but he is taking it, you know, he is doing a lot of thinking” (Man, language advisor, 31 years old, interview in English). Additionally, personal and interpersonal problems were often central in respondents’ explanations and illustrations of “thinking a lot”, and were examples of common content of intrapersonal reflection.

Respondents described that one could easily see when a person was “thinking a lot”, especially when you knew that person well.

You are sitting very quietly and you are thinking and or you sit like this [hands supporting his head] and then you make a [deep sigh], like we call it “sug” (sigh) in Afrikaans. And then they ask you “why are you doing that?” And then you say: “ti |x’an n|a” te (I think a lot). And then someone immediately picks up that yeah something is not right. (Man, language advisor, 35 years old, interview in English)

Non-verbal signals such as sighing, supporting their head with their hands, and sad or angry facial expressions were mentioned as indications that a person was “thinking a lot”. Respondents also mentioned behavioral changes that focused generally on changes in social interactions such as not talking much, not laughing at jokes, not being as friendly as usual, or being absent-minded. Social responses to these signals illustrate that “thinking a lot” is not merely indicative of a cognitive process of rumination, but more indicative of a problematic situation.

### Content and Causes of “Thinking a Lot”

Respondents often described the content of “thinking a lot” in their initial associations and elaborated when we probed for causes of “thinking a lot”. As we began to notice this pattern (after a few interviews), we added questions about possible circumstances under which “thinking a lot” occurred more easily. Many of the respondents were unable to answer the question, others repeated or further elaborated on their first answer. Two respondents mentioned additional causes for “thinking a lot”: (1) a person’s ability to cope with life problems since some individuals may be more prone to “think a lot” and (2) a structural lack of activities and work to keep people occupied. This suggests that although there may be particular circumstances that increase vulnerability to “think a lot”, it is generally understood to be *caused* by a person facing a problem, this is also the *content* of “thinking a lot”.

Respondents mentioned a variety of content that a person could “think a lot” about, which we grouped as personal and interpersonal problems. Personal problems generally consisted of socio-economic difficulties, but also included health concerns, such as HIV/AIDS. Socio-economic difficulties were described as struggles to secure basic needs (e.g. food and clothing) and a need to improve life’s material aspects (e.g. improved housing). Thinking about these issues was considered particularly problematic when there were few opportunities to end these struggles. Ruminating about adversity and experiencing a lack of opportunities or solutions can result in feelings of hopelessness:

Jy moet stres, en dan hoe gaan dit vir jou reg wees, of hoe sal jy maak dat vir jou reg is, so ja… mm…Of jy dink ook: ek gaan net so sterf. Want ek kry nooit iets reg nie. Jy kan ook so dink.

[It can make you stress, and then how is it going to be alright for you, or how will you make it so that it’s alright with you, so yes… or you think: I’m going to die just like this. Because I never get something right (nothing good ever happens to me). You can think like that]. (Woman, 31 years old, interview in Afrikaans)

In Platfontein, fragile economic conditions and lack of employment opportunities are a concern for many people. During informal conversations, uncertainty and a lack of opportunities arose as people complained about unfulfilled governmental promises; the poor maintenance of the main tarred road, poor housing and sanitation; and a general lack of development and employment in the community. Respondents compared the situation in Platfontein with other townships near Kimberley and concluded that they did not receive the same quality and quantity of services. At times, they connected this to their “Bushman” identity as they explained how they (and “Bushman” in general) had been treated unfairly in the past. Other power structures, such as the community leadership (Community Property Association), were blamed for mismanagement and an unequal distribution of resources and opportunities, which added to feeling powerless and mistreated.

Intrapersonal reflections on socio-economic circumstances also had positive connotations as respondents associated “thinking a lot” with learning from mistakes, planning for the future, and setting personal goals. “Thinking a lot” was therefore also associated with constructive and positive reflections.

It can be bad, and it can also be good. Because if you are faced with… we are faced with different situations every day in life… so when I am thinking about a good thing, in a good manner in a good way, it will be good for me. For example when I am thinking about my future, I sit down and keep quiet, just keep to myself, and think what will I want to achieve in life. […] But if it is bad thing, then it won’t be good for me because I’ll be hurting inside, full of emotions, I’ll be keeping in myself, not enjoying life to the fullest … yeah. (Man, 22 years old, interview in English)

A wide range of interpersonal problems were mentioned with main categories as: relationship problems, missing or losing loved ones, and feeling mistreated. “There are a lot of things that will make you ‘think too much.’ Especially at the family, if you fight with your parents or if somebody spread a lie about you… or someone passed away in your family, then you will ‘think too much’” (Woman, 23 years old, interview in Khwe). Respondents further mentioned how “thinking a lot” may be centered on regretting past actions (e.g. saying something bad about someone) and worrying or being concerned about other people (e.g. a parent worrying about the safety of their children).

### Consequences of “Thinking a Lot”

Consequences of “thinking a lot” were described in great variety as primarily negative, but also as neutral. Negative consequences of “thinking a lot” included emotional and psychological problems, social withdrawal, behavioral changes, and somatic complaints. Respondents described emotional and psychological consequences in terms of sadness, loneliness, “hurting inside”, worrying, stressing, losing self-worth, and suicidal thoughts. Some of these states are intricately linked to the life problems the people are thinking about. For example, a person might experience sadness when thinking about a relative who passed away. Rumination about such events may bring about and reinforce such emotions. Emotional and psychological consequences were also described as affecting a person’s life on a deeper level, beyond feeling sad or worrying about a particular situation. One respondent described how “thinking a lot” caused him to focus on negative aspects in life and being unable to get his thinking under control:

You know what happens with me sometimes, when I am negatively affected then I think, you know, my thinking goes to that level whereby, you know, my brain also starts to think negative things. Yesterday, you know, I told people in our house that I really need to get this thing controlled because now it is starting to affect me very negatively. I was not like this before, but now uh I am now, you know, gripped by a lot of negativities. And because I think too much and I give a lot of time in thinking things that are not constructive that are just very destructive. I am like thinking, and I am saying to myself “why am I failure? You know, why don’t I do this? And why…”, you know, things like this. (Man, language advisor, 31 years old, interview in English)

Suicidal thoughts were described as another form of severe affect. A respondent reflected on an event in which someone committed suicide after “thinking a lot” about the death of a close relative:

There was a guy who hang himself because of… he and his cousin… they grow up together. And the other one go to the farm, and then that one was been killed by the other people at the farm. […] And the other one, he was in Platfontein, so then the body of that one was bring to Platfontein and he was buried. And after two days… because that guy he was only alone at his place and he was thinking too much, and then he tell the people “oh, I want to wash myself”. And then normally what we do is, the other people are outside and then he is alone inside. And that time he just hang himself. When they opened the door he is dead. (Man, language advisor, 35 years old, interview in English)

Social withdrawal was mentioned as consequence of “thinking a lot” and described to result from a general lack of interest in social interaction, being too focused on thinking, or because people feel as though other people don’t care about them. Social withdrawal may in turn aggravate the situation because social interaction and talking about problems were considered invaluable for resolving “thinking a lot”. Not talking about the problem is considered to intensify the thinking process and thereby exhaust the brain and consequently cause “madness”:

If you think too much and then there is sometimes people who don’t want to speak about what they think, they just think think, think you know. And then I think sometimes your brain will, it will get tired because you are always using them to think, think, think, and then I think you will go mad. (Woman, 26 years old, interview in English)

Consequences in terms of behavioral changes included being unfriendly, having increased irritability (e.g. shouting, swearing, overreacting, and being bad-tempered), and doing ‘bad’ things (e.g. drugs, drinking alcohol and violent behavior).

Respondents also mentioned a variety of physical consequences of “thinking a lot”, such as a loss of energy, eating less or nothing at all and as a result losing weight, damage to the brain and as a result become *tcó*-*áa* (“mad”) or dying, cardio-vascular complaints such as increased heartbeat, high blood pressure, and having a heart attack, and other bodily complaints such as headache and stomachache.

Dit voel of, jy weet, jy dink en dink, jy bly dink… dat jy… baie goeters, klomp goeters dink, vir my, ek weet nie, as ek klomp goeters dink, dan voel dit vir my of my hart gaan staan … stop. Of ek voel dat my… dinges gaan uitblaas, my kop of…ja… ek voel pyn in my hart

[It feels as if, you know, you think and think, you keep thinking… that you… many things, a whole lot of things. For me, I don’t know, if I’m thinking about a lot of things, then it feels to me as if my heart is going to stop. Or I feel that my… things will burst out, my head or… ja… I feel pain in my heart]. (Woman, 31 years old, interview in Afrikaans)

The embodied experience of “thinking a lot” and catastrophic thoughts indicate that “thinking a lot” is primarily located in the brain or mind and associated with the heart.^10^ The constant rumination of the brain or mind is thought to require a lot of energy. This causes a person to experience a weak feeling in the body and may cause damage to the brain or mind and consequently cause ‘madness’. A high blood pressure, increased heartbeat, and fear of suffering a heart attack are interrelated and reflect the idea that the body is working hard, similar to the brain or mind.

To assess the impact on daily functioning respondents were asked whether “thinking a lot” influences the ability to perform daily tasks. Some respondents mentioned that “thinking a lot” affects daily functioning, but usually in a minor way such as not being able to perform up to usual standards, being forgetful, doing things slowly, or making mistakes. Other respondents mentioned a lack of motivation to do anything and not being able to concentrate at all, which made it extremely difficult to perform daily tasks. And finally, some respondents mentioned that daily tasks or work distract a person from thinking about their problems and therefore has a positive effect, although only temporary.

It is evident that the experience of “thinking a lot” covers a broad range of situations or conditions in everyday life ranging from experiences without negative effects or experiences with mild consequences to experiences with severe consequences. Various factors influence consequences including: personal differences in coping, severity and nature of the problem, ability to talk about the problem, possibility to find a solution or get help, and the duration of “thinking a lot”. “If you think every day, it will be worse, then you will get a heart attack and you will die” (Woman, 79 years old, interview in Khwe). The duration of “thinking a lot” (from an hour to a lifetime), depended on the person’s ability to cope, whether solutions were available, and the severity of the problem: “Maybe you will get somebody [to] tell you: ‘your mum is dead.’ It will take for you a year, it will take for you a long long [time]. But when you have… your relationship is broken. You will never take it so long” (Man, 22 years old, interview in English).

### Coping in Times of “Thinking a Lot”

Respondents mentioned a variety of strategies to stop or find relief from “thinking a lot”: social support, distraction, and religion. Social support became evident in descriptions about the use of Khwe phrases for “thinking a lot” in everyday life. Respondents described situations in which symptoms of “thinking a lot” were picked up and people asked what was bothering the person.

A few days ago I was with a friend and we were sitting and we were chatting and laughing, and that person was quiet for a while, and then the sister asked, “what is wrong? Why are you so quiet?” And then the she said she is thinking too much. Then we asked, “what is wrong?” Then she said she is thinking about what happened two weeks ago [a family member was murdered]. (Man, 26 years old, interview in English)

Sharing problems was considered to be an important strategy to manage “thinking a lot”. To underline its importance, respondents explained that “thinking a lot” would persist and the consequences would be more severe (e.g. committing suicide, having a heart attack) when a person didn’t talk about the problem. “If you are not talking, you are just thinking, then it will be too heavy for you and then in the end it will kill you” (Man, 26 years old, interview in English). Talking about problems was described as having several positive effects. First, sharing and talking about the problem may help a person to “feel relieved”. “If he is just listening to you, then you are opening up… that pain that you have been holding inside, you are taking it out of there. They are just listening to you, then you will find yourself feeling relieved, as if something, a burden has been taking off from you. Yeah” (Man, 22 years old, interview in English). Second, talking about problems opens up opportunities for people to find assistance and a solution for the problem. “If I have problem then I have to speak to someone, openly speak to him and then I get advice from him, so then I can see what I can do” (Man, language advisor, 35 years old, interview in English). And third, people may give advice to refrain from “thinking a lot”. “When I am with my friends they said that I should stop ‘thinking too much’ and let me live my life, this world have their own things and things happens for a reason. So she [the respondent] said that her friends said that I should forget about the problem and continue with my life” (Woman, 41 years old, interview in Khwe).

Another oft-discussed strategy to stop “thinking a lot” was to distract oneself by participating in social activities, singing, reading, listening to music, watching TV, and doing chores or work. Respondents described it as effective, but temporary relief, from “thinking a lot”.

Dan staan ek op and gaan na die mense toe, om te gaan rus, laat afkoel. Ja, ek gaan na die mense, ek sit waar hulle gesels en lag, gaan gesels en lag, en daai goeters moet verby gaan, maar ek, ek los dit nie in. Ek bêre dit. Al gaan ek terug of om te kom slaap, dan begin ek weer daai goeters dink.

[Then I get up and go to the people, to go and rest, cool off. Yes, I go to the people, I just go where they chat and laugh, go and chat and laugh, so that those things must go away/go past, but I, I leave it in here. I hide it away. If I go back (home) to sleep, then I start again to think those things]. (Woman, 31 years old, interview in Afrikaans)

Religion was considered an important source of strength for religious people. Platfontein has many active Christian church groups, many of which are part of the Nederduits Gereformeerde Kerk (Dutch Reformed Church). Respondents explained how religion helped them to manage “thinking a lot” by praying, asking God for strength, and placing their fate and their problems in “God’s hands”. “I don’t tell people what I think about, I just keep it to myself and I prayed to God. And I said that ‘God, you will give me the answer, why did I think this?’ So I didn’t tell someone but I always talk, if I think about my son or brother [who both passed away] I leave it to prayer” (Woman, 49 years old, interview in Khwe).

In addition to the aforementioned strategies, respondents described how their willpower enabled them to refrain from “thinking a lot”. Motivation to decide to stop “thinking a lot” came from experiencing negative effects, seeing how it affected others, or because they realized there was nothing they could do about the problem. One respondent described how he protects himself from “thinking a lot” by refraining from thinking about things that are out of reach.

Soos ek hier sit, ek dink nie aan klomp goeters nie, ek dink net aan iets wat ek wil doen, ja, as ek nou aan iets dink miskien ek will nou iets koop, miskien soos ‘n bicycle – dan moet ek dit doen. Ek kan nie nou, dinge dat dit nou vir my laat kom stres maak, jy sien. As ek nou ken, ek het nie geld om daai ding te bekostig nie, dan kan ek nie aan dink nie, dis hoekom

[As I’m sitting here (as I see life now), I don’t think about a whole lot of things, I just think about something that I want to do, yes, if I think about something, maybe now I want to buy something, like a bicycle – then I must do it. (I am a person that if I think about something, I will act on that – not just think about it) I can’t now allow things about that (plan) to make me all stressed (I don’t let that plan stress me out), you see. If I know now, I don’t have money to afford that thing, then I don’t keep thinking about it, that’s why]. (Man, 21 years old, interview in Afrikaans)

## Discussion

Findings of this study highlight several local meanings and experiences associated with “thinking a lot” among the Khwe. These findings have implications for cross-cultural psychiatric research and the way in which mental health professionals can use such idioms of distress during interventions. In particular, we argue for a meaning-centered and contextualized approach to understand “thinking a lot” in its socio-cultural context and a resistance to generalization. We also call for caution about positioning the idiom in an illness domain and argue that “thinking a lot” should be understood beyond the individual and include interpersonal, community, and socio-political dimensions. Mental health assessment and interventions should therefore be contextualized, work across disciplines, and incorporate local coping strategies.

We identified the Khwe terms *|x’an n|a te* and *|eu-can|a te*, Afrikaans *dink baie*, and English “thinking too much” as idioms of personal and interpersonal distress. These terms are used interchangeably, depending on the language used. “Thinking a lot” is described as a cognitive process of intrapersonal reflection that is primarily about personal and interpersonal problems and associated with negative consequences. “Thinking a lot” has a communicative function since it is used to indicate a problematic situation and evokes social reactions and facilitates social support. The use of idioms of distress in multiple languages brings forth intriguing questions about how words and meanings travel across languages. *|x’an n|a te* and *|eu-can|a te* may have had a long history in Khwe and only recently been translated in other languages due to interaction with outsiders. *Dink baie* could have been picked up from Afrikaners when the Khwe were involved with the SADF. Or “thinking a lot” could have been absorbed from the English and incorporated into Khwe. Comparison of *|x’an n|a te* and *|eu-can|a te* with idioms in Afrikaans (*dink baie*) and English (“thinking too much”) spoken the regions where the Khwe live, could provide more insights into the origin of the idiom of distress, “thinking a lot”, among the Khwe in Platfontein.

The association of “thinking a lot” with ruminating about life problems and sad events also occurs in other settings (Abas and Broadhead 1997; Hinton, Reis, and de Jong 2015; Kaiser et al. 2014). Likewise, similar to other settings, the overall negative focus of “thinking a lot” is further emphasized by narratives that include terms conveying negative emotional states such as stressing or worrying about a problem (Abas and Broadhead 1997; Avotri and Walters 1999), sadness (Abas and Broadhead 1997; Kaiser et al. 2014; Keys et al. 2012), or hopelessness/desperation (Yarris 2014).

Results indicate that “thinking a lot” among the Khwe covers a broad range of psychological and emotional states that may even include brief moments (a few hours or days) of sadness or worrying that do not repeat and are without severe consequences. However, “thinking a lot” also refers to chronic conditions of sadness and hopelessness with severe consequences such as suicidal ideation. Yang and colleagues (2010) report similar gradations in severity for the Chinese idiom “excessive thinking” based on duration and consequences. In other studies, gradations within one concept are not reported, rather specific characteristics of “thinking a lot”, such as chronicity (Kaiser et al. 2014; Yarris 2014), perceived lack of solutions (Yarris 2014), or not focusing on solutions (Kaiser et al. 2014), are used to distinguish it from other idioms of distress. It therefore seems that in some settings “thinking a lot” is more strictly delineated in terms of severity and in relation to other idioms of distress, while in other settings it is used more dynamically. To gain further insight into the use of “thinking a lot” among the Khwe, it would be interesting to compare it with other local idioms of distress; this is, however, beyond the scope of the current study.

The inclusion of “thinking too much” in the DSM-5 as “Kufungisisa”, the Shona term studied by Patel, Simunyu, and Gwanzura (1995), indicates its origin. However, at the same time, “Kufungisisa” is combined with idioms from other regions that share general characteristics and symptoms. Although “thinking a lot” or similar idioms of excessive thinking may share a set of general characteristics such as described in the introduction, grouping them together creates a false sense of similarity and obscures the local nuances that give an idiom meaning within a particular setting. This false similarity contributes to the transformation of idioms of distress as a *type* of distress or “cultural syndrome” instead of meaning a *language* of distress (de Jong and Reis 2010; Nichter 2010). A contextualized, systematic approach for understanding “thinking a lot”, as undertaken in the current study, may reveal more differences among settings. It would be particularly insightful if studies included community samples in addition to clinical samples. Our community sample revealed a dynamic use of the idiom that goes beyond severe distress states; this is something that could be overlooked when depending on clinical samples. In addition, a linguistic analysis of closely affiliated concepts (e.g. Kaiser et al. 2014; Yarris 2014) could reveal a range of severity across concepts. Moreover, contextualization by focusing on the content of “thinking a lot”, consequences and experiences, and ways of coping should all be included as was done in this study. For example, Hinton, Reis, and de Jong (2015) systematically determined the content of “thinking a lot” in a Cambodian refugee context and thereby gained insight into the ecological context.

The broad range of emotional and psychological states that the Khwe associated with “thinking a lot” includes non-pathological conditions. Respondents’ narratives described brief episodes of “thinking a lot” in which they were able to cope with a stressor through intrapersonal reflection and taking effective measures. In these cases, the “perceived inability to cope” and “harm”, defined by Ridner (2004) as two of the five attributes of psychological distress, were not applicable since respondents’ actions enabled them to manage the episode of “thinking a lot” and prevented emotional, psychological, social and physical harm. The application of an idiom across a broad range of psychological and emotional states is not uncommon. Idioms of distress are described as a way to communicate experiential states of varying severity and are therefore not necessarily indications for psychopathological symptoms (American Psychiatric Association 2013; Nichter 2010). Yet, the general tendency to understand a concept such as “thinking a lot” as psychopathology, for example, by depending on a clinical study sample and comparing it to a mental disorder, obscures its broad range. This may unintentionally pull local idioms into an illness domain.

Abramowitz (2010) cautions about appropriating idioms into clinical settings in the name of cultural sensitivity. This approach may strip the idioms of their meanings and merely function as pidgin psychiatry through which Western classification systems and treatment are implemented. However, idioms of distress are still meaningful in clinical settings in terms of understanding a patient’s life world and the patient’s treatment priorities. Discussing the idiom of distress may reveal life distress, trauma, psychopathology, destructive behavior, negative affective states, the patient’s understanding of disturbances in psychology and physiology, the patient’s attempts at recovery, and thus such and evaluation facilitates consensual treatment negotiation (Hinton and Lewis-Fernández 2010). Caution about the clinical use of “thinking a lot” is still warranted, and, when encountered in clinical settings, should be accompanied with an assessment of severity to distinguish between psychopathological and non-psychopathological states.

Studies on “thinking a lot” may provide useful characteristics to determine the severity (e.g. duration, and psychological, social and physical consequences). These characteristics and their emphasis likely differ among settings as they reflect local ethnopsychology and ethnophysiology. Hinton, Reis, and de Jong (2015) conducted a study in which the salience and severity of cognition and somatic complaints during episodes of “thinking a lot” were systematically assessed among Cambodian refugees. Such an approach facilitates the identification of key symptoms and complaints to be addressed in clinical settings.

“Thinking a lot” should also be understood beyond an individual dimension. Respondents situated “thinking a lot” in a socio-political, economic, and social context. The sources of distress described by respondents reflect their marginalized and displaced position. Much concern was directed at the high unemployment rate: 95 % compared to an average of 22 % in the general population of the Northern Cape and 28 % in South Africa (South African San Institute 2010).^11^ For the Khwe and !Xun, 97 % live on less that 1 US dollar per day compared to 7 % in the Northern Cape and 40 % in South Africa (South African San Institute 2010). Some respondents described a sense of hopelessness and lack of control over circumstances in relation to unemployment and poverty. In the history of the Khwe and !Xun, uncertainty and dependence on structures such as the SADF and South African government were key characteristics that shaped their lives. This is also reflected in the community’s current experience of neglect by the local government. Some community members attributed their marginalization to their “bushman” identity, which further illustrates how their experience is embedded in socio-political dimensions. Results also draw our attention to the central and ambiguous role^12^ of social relationships as source of distress and support. Many respondents described various forms of social support that enabled them to cope with stressors. The pivotal role of social support as a resource to overcome adversities in life is widely acknowledged (Thoits 2011). “Thinking a lot” was, however, also often associated with interpersonal problems and social isolation, which may hinder social support. Displacement is known to have negative effects on social life, such as loss of connection to family and other support networks, and loss of valued social roles and meaningful activities (Miller and Rasco 2004:1–66). The Khwe and !Xun communities consist of family groups and have a long shared history (Den Hertog 2013) and therefore retained a large part of their social support networks. Yet, community and family structures were disrupted in their displacement as people died in violent conflicts and many family members remained behind in Namibia and Angola. In their current situation the Khwe and Xun’s social life continues to be under pressure due to people lost due to HIV/AIDS, relationship tension brought about by alcohol abuse and absence of family members due to employment opportunities that require individuals (mostly men) to stay at distant work locations for periods of three to nine months.

“Thinking a lot” is an idiom of distress, and mental health interventions should assess the local meaning of “thinking a lot” and contextualize it as we have done in the current study. The multi-level dynamics of socio-political, economic, and social context and individual experiential states revealed in this study illustrate that “thinking a lot” should be understood beyond the individual and include interpersonal, community and socio-political dimensions. For mental health interventions, this requires an approach that works across disciplines and sectors, focuses on strengthening social fabric of communities, acknowledges and builds on individual and community strengths, and incorporates local ways of managing “thinking a lot” (de Jong and Komproe 2002; Hinton, Reis, and de Jong 2015; Hinton and Kirmayer 2013; Miller and Rasco 2004:1–66; Saleebey 2000; Summerfield 2000).

## Footnotes

1 ! symbolizes an alveolar click.

2 Throughout history, these labels have been contested, depending on the era and individual opinion they were/are considered derogatory, actively taken up and seen as proud heritage, or somewhere in between. In Platfontein “San” is preferred over “Bushman” (Letsoalo 2010).

3 We are grateful to the Centre for Communication, Media & Society (CCMS) at the University of KwaZulu-Natal, and specifically Prof. Dr. Tomaselli, for facilitating this process.

4 *Dink* translates as “think” and *baie* as “much” or “a lot”.

5 *Dink* translates as “think”, *aan* as “of”, *klomp* “lot”, and *goeters* “things”.

6 | symbolizes a dental click.

7 *|x’an* is translated as “much” and *n|a te* as “thinking”.

8 *|eu-ca* is translated as “big” and *n|a te* as “thinking”.

9 *tiya* is translated as “many”, *xo’a* as “things” and *n|a* as “thinking”.

10 We thank the reviewer for directing our attention these findings.

11 This source refers to data from a Platfontein community survey in 2005 and the Human Development Report of 2006.

12 We thank the reviewer for directing our attention to this theme.
